# Automated methods to test connectedness and quantify indirectness of evidence in network meta‐analysis

**DOI:** 10.1002/jrsm.1329

**Published:** 2018-12-04

**Authors:** Howard Thom, Ian R. White, Nicky J. Welton, Guobing Lu

**Affiliations:** ^1^ Bristol Medical School: Population Health Sciences University of Bristol Bristol UK; ^2^ MRC Clinical Trials Unit University College London London UK

**Keywords:** Connectedness testing, Disconnected network, Graph theory, Indirect comparison, Network meta‐analysis

## Abstract

Network meta‐analysis compares multiple treatments from studies that form a connected network of evidence. However, for complex networks, it is not easy to see if the network is connected. We use simple techniques from graph theory to test the connectedness of evidence networks in network meta‐analysis. The method is to build the adjacency matrix for a network, with rows and columns corresponding to the treatments in the network and entries being one or zero depending on whether the treatments have been compared or not, and with zeros along the diagonal. Manipulation of this matrix gives the indirect connection matrix. The entries of this matrix determine whether two treatments can be compared, directly or indirectly. We also describe the distance matrix, which gives the minimum number of steps in the network required to compare a pair of treatments. This is a useful assessment of an indirect comparison as each additional step requires further assumptions of homogeneity in, for example, design and target populations of included trials. If there are no loops in the network, the distance is a measure of the degree of assumptions needed; it is approximately this with loops. We illustrate our methods using several constructed examples and giving R code for computation. We have also implemented the techniques in the Stata package “network.” The methods provide a fast way to ensure comparisons are only made between connected treatments and to assess the degree of indirectness of a comparison.

## 1 INTRODUCTION

Network meta‐analysis, or mixed treatment comparison, is a statistical method to synthesize comparative evidence on multiple treatments provided by a set of randomized controlled trials (RCTs) that forms a connected network of treatment comparisons.^1^ These networks, or graphs, can be analyzed using the extensive literature of graph theory^2^; this theory has many applications in fields other than network meta‐analysis, including social media,^3^ medicine,^4^ computational chemistry,^5^ and sociology.^6^ An evidence network is a graph that consists of vertices, representing the treatments and edges, representing the available comparisons between pairs of treatments. Vertices corresponding to treatments are said to be adjacent if there is an edge connecting them. Vertices are said to be connected if there is a chain of adjacent vertices connecting them, named a “walk.” If treatments represented by the vertices are connected, they can be compared through a chain of RCT evidence.^7^ A network is connected if every two vertices are connected. A glossary of terms used in this manuscript is provided in Table 1.

**Table 1:** Glossary of terms and symbols

Term/symbol	Explanation
Vertices	Vertices are the nodes in an evidence network corresponding to treatments.
Edge	There is an edge between two vertices if the corresponding treatments were compared in at least one RCT.
Adjacent	Two vertices are adjacent if there is an edge between them. This means the corresponding treatments have been compared in at least one RCT.
Walk	A chain of adjacent vertices in a graph.
Connected	Vertices are connected if there is a walk connecting them. This means that the treatments represented by the vertices can be compared through a chain of evidence.
Adjacency matrix, *A*	An *n* × *n* symmetric matrix, where *n* is the number of treatments, with elements *a*_*ij*_ = 1 if treatments *i* and *j* have been compared in an RCT (that is, if there is direct evidence relating them), and *a*_*ij*_ = 0 otherwise, with zeros on the diagonal.
Indirect connection matrix, *I*(*C*_*n* − 1_)	An *n* × *n* symmetric matrix with *I*(*C*_*n* − 1_)_*ij*_ = 1 if there is a walk between treatments *i* and *j* (that is, if they are connected) and *I*(*C*_*n* − 1_)_*ij*_ = 0 otherwise, with ones on the diagonal.
Distance matrix, *D*	An *n* × *n* symmetric matrix with *d*_*ij*_ being the length of the shortest walk between treatments *i* and *j* and zero if they are not connected, with zeros on the diagonal.

Abbreviation: RCT, randomized controlled trial.

**Figure 1:**
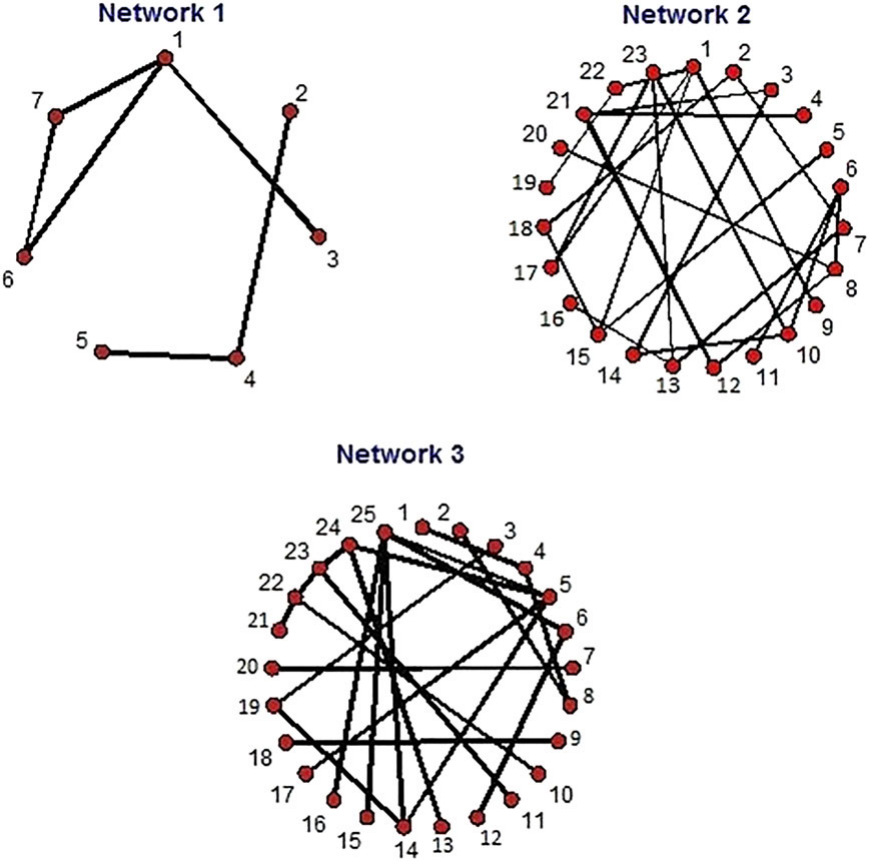
Three example networks of evidence with different degrees of connectedness and complexity [Colour figure can be viewed at wileyonlinelibrary.com]

Figure 1 illustrates several networks, including the complex networks 2 and 3. It is not immediately clear from visual inspection that network 2 is connected while network 3 is not. Visual inspection of evidence networks is time‐consuming and prone to error, especially as there can be many networks in an analysis, covering different time‐points, outcomes, treatment definitions, and subgroup and sensitivity analyses. Automated network connectedness testing methods have been implemented in the R statistical language^8^; these include the breadth‐first search of the “igraph” package and an implementation in “netmeta” using a distance algorithm.^9^, ^10^, ^11^ In this paper, we explain an alternative, matrix‐based method from graph theory, which is a fast and simple method to test connectedness. We propose that the connectedness test should be applied before any network meta‐analysis to quickly exclude disconnected components of a network. We also discuss a distance matrix that provides the degree of separation, or indirectness, between treatments in an evidence network. As trials on different treatments can differ in their design, target population, and other ways, this can be a useful illustration of the assumptions of an indirect comparison.

We begin by describing the methods and then illustrate their application to a range of networks of varying complexity. We have implemented these methods in the Stata package “network”^12^, ^13^ but here provide algorithms for use in other software and code for use in the R statistical language.

## 2 METHODS

### 2.1 The adjacency and indirect connection matrices

We first construct an *n* × *n* symmetric matrix, *A*, which is called the adjacency matrix, where *n* is equal to the number of treatments in the network *G*. We set the element *a*_*ij*_ = 1 if treatments *i* and *j* have been compared in a trial, and *a*_*ij*_ = 0 otherwise. The diagonal is filled with zeros, so *a*_*ii*_ = 0. Note that *G* may have multiple matrices *A* for different orderings of the vertices, but these matrices are equivalent in representing the adjacency relation. We can therefore work with an adjacency matrix A for a given ordering of vertices. Raising A to a power *k* counts the total numbers of *k* step walks connecting vertices corresponding to each row and column. For example, the (1, 2) entry of matrix *A*^3^ would count the number of three‐step walks, called 3‐walks, from treatment 1 to treatment 2. This includes 3‐walks with loops such as 1 → 2 → 3 → 2 as well as nontrivial 3‐walks like 1 → 3 → 4 → 2. In graph theory, nontrivial walks that visit each vertex at most once, are termed “paths.” In a network with *n* treatments, the maximum length of a path is *n* − 1 steps. The (*i*, *j*) entry of the sum of the powers of the adjacency matrix to *l*

$$C_{l}=\sum_{k=1}^{l}A^{k},$$

counts the total number of walks of length *l*, or less, between treatments *i* and *j*. The (*i*, *j*) entry of *C*_*n* − 1_ counts the total number of walks of length *n* − 1, or less, between treatments *i* and *j*. Setting all nonzero elements to 1 gives *I*(*C*_*n* − 1_), which we term the indirect connection matrix, where the *I*() operator sets elements to 1 if they are nonzero and zero otherwise. If all off diagonal elements of *I*(*C*_*n* − 1_) are one, then the network is fully connected. Otherwise, the network is disconnected, and it is possible to form a block diagonal indirect connection matrix, after some permutation of the row and column indices, indicating that only treatments within each block are connected. Trials with more than two arms contribute additional ones to the adjacency matrix but the indirect connection matrix is still given by *I*(*C*_*n* − 1_) as it only depends on whether or not two treatments have been compared in a trial.

### 2.2 Illustration of method

**Figure 2:**
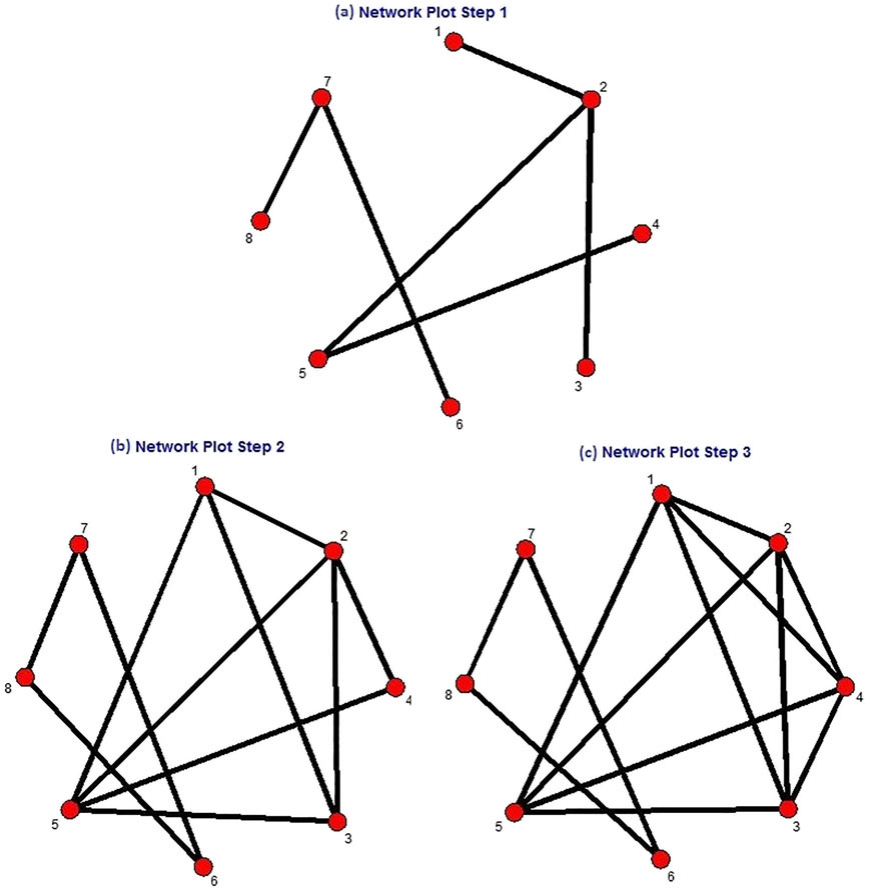
Illustrative evidence network with eight treatments. Each step plots the comparisons possible with that number of steps on the evidence network [Colour figure can be viewed at wileyonlinelibrary.com]

Consider the network illustrated in Figure 2A, which was generated using a freely available routine^14^ within Stata. This consists of eight hypothetical treatments labeled 1 through 8. The evidence consists of six pairwise RCTs, represented as six edges in the network, comparing 1 with 2, 2 with 3, 2 with 5, 4 with 5, 6 with 7, and 7 with 8. The adjacency matrix is therefore

$$A=\left(\begin{array}{cccccccc} 0 & 1 & 0 & 0 & 0 & 0 & 0 & 0\\ 1 & 0 & 1 & 0 & 1 & 0 & 0 & 0\\ 0 & 1 & 0 & 0 & 0 & 0 & 0 & 0\\ 0 & 0 & 0 & 0 & 1 & 0 & 0 & 0\\ 0 & 1 & 0 & 1 & 0 & 0 & 0 & 0\\ 0 & 0 & 0 & 0 & 0 & 0 & 1 & 0\\ 0 & 0 & 0 & 0 & 0 & 1 & 0 & 1\\ 0 & 0 & 0 & 0 & 0 & 0 & 1 & 0 \end{array}\right),$$

where an entry *a*_*ij*_ is 1 if treatments *i* and *j* have been compared in at least one RCT and zero otherwise.

*A*^2^ indicates the number of 2‐walks between each pair of treatments. We need only know *A* to obtain *A*^2^, and that *A*^2^ counts the number of 2‐walks is made intuitive by considering the treatment connections implied by *A*. For example, the first row of *A* indicates which treatments are directly connected to treatment 1. Multiplying row 1 by the *j*th column tells us where walks can go in two steps from 1 via *j*. In the first row of this example, all entries other than the 2nd are zero, so only treatments connected to treatment 2, indicated by the second row, will contribute to the calculation. Treatment 2 is connected to 1, 3, and 5, and so the first row of *A*^2^ has 1 in these entries. The same process is repeated to obtain all rows of *A*^2^.

This 2‐walk matrix is

$$A^{2}=\left(\begin{array}{cccccccc} 1 & 0 & 1 & 0 & 1 & 0 & 0 & 0\\ 0 & 3 & 0 & 1 & 0 & 0 & 0 & 0\\ 1 & 0 & 1 & 0 & 1 & 0 & 0 & 0\\ 0 & 1 & 0 & 1 & 0 & 0 & 0 & 0\\ 1 & 0 & 1 & 0 & 2 & 0 & 0 & 0\\ 0 & 0 & 0 & 0 & 0 & 1 & 0 & 1\\ 0 & 0 & 0 & 0 & 0 & 0 & 2 & 0\\ 0 & 0 & 0 & 0 & 0 & 1 & 0 & 1 \end{array}\right).$$

Note that all treatments can be connected to themselves by at least one 2‐walk. For example, treatment 2 has three 2‐walks looping back to itself, namely 2 → 1 → 2, 2 → 3 → 2, and 2 → 5 → 2. Figure 2B shows the comparisons that can be made using walks of one or two steps and is the network corresponding to adjacency matrix *I*(*C*_2_). This network directly connects any treatment from the network in Figure 2A that can be compared in one or two steps.

*A*^3^ indicates the number of 3‐walks between treatments.

$$A^{3}=\left(\begin{array}{cccccccc} 0 & 3 & 0 & 1 & 0 & 0 & 0 & 0\\ 3 & 0 & 3 & 0 & 4 & 0 & 0 & 0\\ 0 & 3 & 0 & 1 & 0 & 0 & 0 & 0\\ 1 & 0 & 1 & 0 & 2 & 0 & 0 & 0\\ 0 & 4 & 0 & 2 & 0 & 0 & 0 & 0\\ 0 & 0 & 0 & 0 & 0 & 0 & 2 & 0\\ 0 & 0 & 0 & 0 & 0 & 2 & 0 & 2\\ 0 & 0 & 0 & 0 & 0 & 0 & 2 & 0 \end{array}\right).$$

For example, the first row of *A*^3^ indicates that treatment 1 is connected to treatments 2 and 4 in walks of three steps. The matrix *C*_3_ is the sum of *A*^1^ + *A*^2^ + *A*^3^ and this gives

$$I\left(C_{3}\right)=\left(\begin{array}{cccccccc} 1 & 1 & 1 & 1 & 1 & 0 & 0 & 0\\ 1 & 1 & 1 & 1 & 1 & 0 & 0 & 0\\ 1 & 1 & 1 & 1 & 1 & 0 & 0 & 0\\ 1 & 1 & 1 & 1 & 1 & 0 & 0 & 0\\ 1 & 1 & 1 & 1 & 1 & 0 & 0 & 0\\ 0 & 0 & 0 & 0 & 0 & 1 & 1 & 1\\ 0 & 0 & 0 & 0 & 0 & 1 & 1 & 1\\ 0 & 0 & 0 & 0 & 0 & 1 & 1 & 1 \end{array}\right).$$

Figure 2C shows the treatments that can be compared in one, two, or three steps and is the network corresponding to adjacency matrix *I*(*C*_3_).

We now apply the same blocking (rows/columns 1–5, rows/columns 6–8) to *A* as we do to *I*(*C*_3_) and it becomes clear that *A* also has a block diagonal structure, with entries outside the two corner blocks zero. Further powers will therefore remain block diagonal.^15^ As a sum of block diagonal matrices is also block diagonal, *I*(*C*_3_), with its blocks completely filled, will be identical to *I*(*C*_7_), the indirect connection matrix.

$$I\left(C_{7}\right)=\left(\begin{array}{cccccccc} 1 & 1 & 1 & 1 & 1 & 0 & 0 & 0\\ 1 & 1 & 1 & 1 & 1 & 0 & 0 & 0\\ 1 & 1 & 1 & 1 & 1 & 0 & 0 & 0\\ 1 & 1 & 1 & 1 & 1 & 0 & 0 & 0\\ 1 & 1 & 1 & 1 & 1 & 0 & 0 & 0\\ 0 & 0 & 0 & 0 & 0 & 1 & 1 & 1\\ 0 & 0 & 0 & 0 & 0 & 1 & 1 & 1\\ 0 & 0 & 0 & 0 & 0 & 1 & 1 & 1 \end{array}\right).$$

As expected, this matrix tells us that treatments 1 through 5 can be directly or indirectly compared with each other but not to treatments 6, 7, or 8. These three treatments can only be compared, directly or indirectly, with each other.

The appendix provides a proof for the interested reader and R code to calculate the matrix for networks of arbitrary complexity. The input data is the treatment matrix in the format used by the National Institute for Health and Care Excellence Decision Support Unit Technical Support Documents.^16^, ^17^ We have implemented these methods in the Stata package network, which routinely computes this matrix when a network is set up and issues a warning if the network is disconnected^12^; this is also described in the appendix.

### 2.3 The distance matrix

The distance matrix of an evidence network represents the length of the shortest path between two vertices and represents a more informative alternative to the indirect connection matrix. These matrices are symmetric and can be arranged to be block diagonal if the evidence network splits into disconnected components. Note that it is useful to keep track of the treatment labels of the rows and columns in rearranged matrices, and we do so in our applications below. The main diagonal is zero as the distance between a vertex and itself is zero. For the network in Figure 2A, the distance matrix is

$$D=\left(\begin{array}{cccccccc} 0 & 1 & 2 & 3 & 2 & 0 & 0 & 0\\ 1 & 0 & 1 & 2 & 1 & 0 & 0 & 0\\ 2 & 1 & 0 & 3 & 2 & 0 & 0 & 0\\ 3 & 2 & 3 & 0 & 1 & 0 & 0 & 0\\ 2 & 1 & 2 & 1 & 0 & 0 & 0 & 0\\ 0 & 0 & 0 & 0 & 0 & 0 & 1 & 2\\ 0 & 0 & 0 & 0 & 0 & 1 & 0 & 1\\ 0 & 0 & 0 & 0 & 0 & 2 & 1 & 0 \end{array}\right).$$

This matrix describes the degree of indirectness of the evidence used to compare treatments in evidence networks. Higher entries represent more indirect evidence. For example, it requires three steps to compare treatments 1 and 4 but only 2 to compare treatments 1 and 3. Also, the matrix tells us that treatments 1 and 2 can be compared directly as there is only one step between them.

The distance matrix can be calculated from the sums of the powers of the adjacency matrix *A* via the formula

$$D=A+\sum_{i=2}^{n-1}i\left(I\left(C_{i}\right)-I\left(C_{i-1}\right)\right)$$

Code to calculate the distance matrix using this formula is provided in the appendix.

### 2.4 Collecting matrices into connected components

We suggest collecting the indirect connection and distance matrices into block diagonal format where each block represents a connected subnetwork. These block diagonal matrices make it easy to see how many connected components there are in the network. If the matrices consist of a single block, then the entire network is connected. We provide code for a simple recursive sorting function in the appendix.

## 3 APPLICATION TO EXAMPLE NETWORKS

We apply our connectedness test to a series of evidence networks to illustrate the utility and behavior of the algorithm. The examples are illustrated in Figure 1.

Consider network 1 in Figure 1. In this case, there are seven treatments and six direct pairwise comparisons. The adjacency matrix is presented in Table 2 and the indirect connection matrix is presented in Table 3. The distance matrix with connected components grouped to form a block diagonal matrix is presented in Table 4. The collected matrix allows us to quickly see which treatments can be compared and that there are two connected subcomponents. For example, treatments 1, 3, 6, and 7 can be compared and form a connected subcomponent but cannot be compared with 2, 4, and 5, which form another connected subcomponent. The distance matrix quickly summarizes the additional information that no treatment is more than two steps from any to which it is connected and that overall most connections are direct (single step) comparisons.

**Table 2:** Adjacency matrix for network 2

	*1*	*2*	*3*	*4*	*5*	*6*	*7*
*1*	0	0	1	0	0	1	1
*2*	0	0	0	1	0	0	0
*3*	1	0	0	0	0	0	0
*4*	0	1	0	0	1	0	0
*5*	0	0	0	1	0	0	0
*6*	1	0	0	0	0	0	1
*7*	1	0	0	0	0	1	0

**Table 3:** Indirect connection matrix for network 2

	1	2	3	4	5	6	7
1	1	0	1	0	0	1	1
2	0	1	0	1	1	0	0
3	1	0	1	0	0	1	1
4	0	1	0	1	1	0	0
5	0	1	0	1	1	0	0
6	1	0	1	0	0	1	1
7	1	0	1	0	0	1	1

**Table 4:** Distance matrix for network 2 collected into connected subcomponents

	1	3	6	7	2	4	5
1	0	1	1	1			
3	1	0	2	2			
6	1	2	0	1			
7	1	2	1	0			
2					0	1	2
4					1	0	1
5					2	1	0

Network 2 in Figure 1 consists of 23 treatments and 25 direct pairwise comparisons. The collected distance matrix is presented in Table 5 and tells us everything the indirect connection matrix would. This quickly shows that the network is completely connected as it consists of a single block that includes all treatments. The distance matrix also tells us that many of the comparisons, such as that between treatments 4 and 5, rely on nine steps along the network and are thus quite indirect. This warns us that many assumptions of homogeneity are necessary to compare such treatments. It is also a warning that we are close to disconnectedness, although this depends on the strength of evidence. A high valued distance matrix indicates a sparse network that would be more likely to become disconnected if treatments were split further, for example, by dose or frequency, or if a subset of trials were to be removed for subgroup analysis.

**Table 5:** Distance matrix for network 2. The network is connected so collected matrix consists of single block

	1	2	3	4	5	6	7	8	9	10	11	12	13	14	15	16	17	18	19	20	21	22	23
1	0	3	5	7	2	4	4	5	1	3	5	6	3	4	1	4	1	2	2	6	6	1	2
2	3	0	6	8	3	5	1	6	4	4	6	7	2	5	2	3	4	1	5	7	7	4	3
3	5	6	0	2	7	3	5	3	6	2	4	2	4	1	6	5	4	7	7	4	1	6	3
4	7	8	2	0	9	4	7	3	8	4	5	2	6	3	8	7	6	9	9	4	1	8	5
5	2	3	7	9	0	6	4	7	3	5	7	8	5	6	1	6	3	2	4	8	8	3	4
6	4	5	3	4	6	0	4	1	5	1	1	2	3	2	5	4	3	6	6	2	3	5	2
7	4	1	5	7	4	4	0	5	5	3	5	6	1	4	3	2	3	2	6	6	6	5	2
8	5	6	3	3	7	1	5	0	6	2	2	1	4	3	6	5	4	7	7	1	2	6	3
9	1	4	6	8	3	5	5	6	0	4	6	7	4	5	2	5	2	3	3	7	7	2	3
10	3	4	2	4	5	1	3	2	4	0	2	3	2	1	4	3	2	5	5	3	3	4	1
11	5	6	4	5	7	1	5	2	6	2	0	3	4	3	6	5	4	7	7	3	4	6	3
12	6	7	2	2	8	2	6	1	7	3	3	0	5	3	7	6	5	8	8	2	1	7	4
13	3	2	4	6	5	3	1	4	4	2	4	5	0	3	4	1	2	3	5	5	5	4	1
14	4	5	1	3	6	2	4	3	5	1	3	3	3	0	5	4	3	6	6	4	2	5	2
15	1	2	6	8	1	5	3	6	2	4	6	7	4	5	0	5	2	1	3	7	7	2	3
16	4	3	5	7	6	4	2	5	5	3	5	6	1	4	5	0	3	4	6	6	6	5	2
17	1	4	4	6	3	3	3	4	2	2	4	5	2	3	2	3	0	3	3	5	5	2	1
18	2	1	7	9	2	6	2	7	3	5	7	8	3	6	1	4	3	0	4	8	8	3	4
19	2	5	7	9	4	6	6	7	3	5	7	8	5	6	3	6	3	4	0	8	8	1	4
20	6	7	4	4	8	2	6	1	7	3	3	2	5	4	7	6	5	8	8	0	3	7	4
21	6	7	1	1	8	3	6	2	7	3	4	1	5	2	7	6	5	8	8	3	0	7	4
22	1	4	6	8	3	5	5	6	2	4	6	7	4	5	2	5	2	3	1	7	7	0	3
23	2	3	3	5	4	2	2	3	3	1	3	4	1	2	3	2	1	4	4	4	4	3	0

Consider the complex network 3 in Figure 1, which has 25 treatments and 22 direct pairwise comparisons. The collected distance matrix presented in Table 6 shows that there are four connected components and shows the treatments in each component. In network 3, we might be especially interested in comparisons with treatment 1 and we could use the indirect connection matrix to omit treatments from the network that are not connected to 1, in this case, all but treatments 2, 4, and 8. Simplifying the network by removing disconnected components can save computational time. More importantly, it removes the danger and distraction of interpreting disconnected comparisons. In addition to this utility, the distance matrix summarizes the number of steps between any two treatments. In this case, the maximum number of steps is seven between treatments 3 and 10, 10 and 12, 3 and 21, and 12 and 21; this suggests that these rely on many assumptions and are more likely to become disconnected if treatment definitions are changed or RCTs omitted. In conjunction with uncertainty intervals and probabilities of superiority and inferiority, this matrix can help to interpret the strength of the conclusions of a network meta‐analysis. Even if the statistical uncertainty in the comparison between treatment 3 and 10 is low, for example, we may not be as confident in the results if the comparison was based on seven indirect steps, rather than on a single step, which would be direct evidence. However, the distance matrix does not tell us about the precision or quality of the evidence along walks.

**Table 6:** Distance matrix for network 3 collected into connected subcomponents

	1	2	4	8	3	5	6	10	11	12	13	14	15	16	17	19	21	22	23	24	25	7	20	9	18
1	0	3	1	2																					
2	3	0	2	1																					
4	1	2	0	1																					
8	2	1	1	0																					
3					0	3	4	7	6	5	5	2	4	4	4	1	7	6	5	4	3				
5					3	0	2	4	3	3	2	1	2	2	1	2	4	3	2	1	1				
6					4	2	0	6	5	1	4	2	2	2	3	3	6	5	4	3	1				
10					7	4	6	0	3	7	4	5	6	6	5	6	2	1	2	3	5				
11					6	3	5	3	0	6	3	4	5	5	4	5	3	2	1	2	4				
12					5	3	1	7	6	0	5	3	3	3	4	4	7	6	5	4	2				
13					5	2	4	4	3	5	0	3	4	4	3	4	4	3	2	1	3				
14					2	1	2	5	4	3	3	0	2	2	2	1	5	4	3	2	1				
15					4	2	2	6	5	3	4	2	0	2	3	3	6	5	4	3	1				
16					4	2	2	6	5	3	4	2	2	0	3	3	6	5	4	3	1				
17					4	1	3	5	4	4	3	2	3	3	0	3	5	4	3	2	2				
19					1	2	3	6	5	4	4	1	3	3	3	0	6	5	4	3	2				
21					7	4	6	2	3	7	4	5	6	6	5	6	0	1	2	3	5				
22					6	3	5	1	2	6	3	4	5	5	4	5	1	0	1	2	4				
23					5	2	4	2	1	5	2	3	4	4	3	4	2	1	0	1	3				
24					4	1	3	3	2	4	1	2	3	3	2	3	3	2	1	0	2				
25					3	1	1	5	4	2	3	1	1	1	2	2	5	4	3	2	0				
7																						0	1		
20																						1	0		
9																								0	1
18																								1	0

## 4 DISCUSSION

We have presented a test of connectedness of networks that is easy to automate and can be applied to any network meta‐analysis with trials with any number of arms. We recommend applying this test as a preliminary step, which can be incorporated within software, to conduct a network meta‐analysis. Our method can quickly inform the analyst about the number of connected components of a network and what comparisons are possible. If disconnected treatments are compared, results may be numerically unstable and variances high, although high variances are themselves a warning that treatments are disconnected. Without an explicit test, analysts may work out probabilities of ranks or put the effect estimates into cost‐effectiveness analyses without realizing their comparisons are invalid. This is a greater concern when many networks (eg, different time points, outcomes, or subgroups) are analyzed as it is more likely a disconnected network will be missed if connectedness is tested manually.

We have also presented the distance matrix, which we recommend presenting alongside the results of an analysis. The distance matrix quantifies how indirect the evidence on each comparison is, which is of interest as every additional step requires further assumptions about heterogeneity of the trial designs and populations. High values in the distance matrix also provide a warning that further splitting of treatments or exclusion of trials are more likely to lead to a disconnected network. We do not recommend downgrading comparisons that are more indirect, only paying greater attention to the necessary assumptions of heterogeneity along the walks for such comparisons. As the distance matrix includes all the information of the indirect connection matrix, we recommend presenting only the distance matrix. Alongside standard errors and risk of bias assessments, it can be a useful tool to judge our confidence in the results.

Graph theory has recently been applied to network meta‐analysis to ease visualization and analysis. One application is to rearrange the vertices of a network to avoid edges that overlap and simplify network plots.^18^, ^19^ Using these rearrangements, it is easier to see which components of the network are connected, and the netmeta package has implemented connectedness testing using an alternative algorithm.^9^, ^11^ Inconsistency between direct and indirect evidence can arise if there are loops in the evidence network that are not entirely due to a single multi‐arm trial.^20^ Graphical methods have been proposed to identify hotspots of potential inconsistency in the network but these need human judgment for further testing.^21^ A recent application of graph theory was to automatically identify potential inconsistencies to be assessed by node‐splitting.^22^ We believe there are many further such uses for graph theory in network meta‐analysis.

One possible application would be to use graph theoretic methods to assess the strength of evidence on each comparison, perhaps combining our distance matrix with sample sizes, variance estimates, and risk of bias assessments. The contributions matrix of Krahn et al^21^ also implemented in the netmeta package,^11^ assesses the contribution of each trial design to each comparison but does not assess the absolute strength of evidence or inconsistency. The distance matrix only assesses indirectness of each comparison. It may be possible to assess the strength of each of the edges in a walk and give a single overall assessment of the evidence informing each comparison. However, this may not give additional information on top of the results of the network meta‐analysis itself.

In summary, we have presented computationally efficient techniques to assess the connectedness and indirectness of evidence networks. We believe these methods will help simplify the practice and presentation of network meta‐analysis in the future.

## CONFLICTS OF INTEREST

The authors have no conflicts of interest to report.

## APPENDIX A

### PROOF OF CONNECTEDNESS TEST

We prove that treatment vertices *i* and *j* are connected directly or indirectly if and only if the entry *I*(*C*_*n* − 1_)_*ij*_ is nonzero. Let G be a network with vertices *v*_1_, …, *v*_*n*_ and *A* its adjacency matrix with *a*_*ij*_ = 1 if *v*_*i*_ and *v*_*j*_ (*i* ≠ *j*) are adjacent and 0 otherwise. The diagonal elements *a*_*jj*_ = 0, ie, *v*_*j*_ is regarded not to be connected to itself.

Theorem 1: For any integer *k*, the (*i*, *j*)‐entry of *A*^*k*^ is equal to the number of *k*‐walks from *v*_*i*_ to *v*_*j*_.
Proof: We use mathematical induction. Let $a_{\mathrm{ij}}^{\left(k\right)}$ be the (*i*, *j*)‐entry of the matrix *A*^*k*^. Theorem 1 holds obviously for *A*,i.e., *k* = 1. Suppose that it holds for *A*^*k*^, then because *A*^*k* + 1^ = *AA*^*k*^, we have

$$a_{\mathrm{ij}}^{\left(k+1\right)}=\sum_{u}a_{\mathrm{iu}}a_{\mathrm{uj}}^{\left(k\right)},$$

where *a*_*iu*_ is the number of all 1‐walks from *v*_*i*_ to *v*_*u*_ and $a_{\mathrm{uj}}^{\left(k\right)}$ is the number of all *k*‐walks from *v*_*u*_ to *v*_*j*_. So $a_{\mathrm{iu}}a_{\mathrm{uj}}^{\left(k\right)}$ is the number of all (*k* + 1)‐walks from *v*_*i*_ to *v*_*j*_ that go through *v*_*u*_, and hence $a_{\mathrm{ij}}^{\left(k+1\right)}$ is the number of all (*k* + 1)‐walks from *v*_*i*_ to *v*_*j*_. That is, Theorem 1 holds for *k* + 1. □.

Then, as a corollary of Theorem 1, we have

Theorem 2: G is a connected evidence network if and only if the matrix

$$C_{n-1}=\sum_{k=1}^{n-1}A^{k},$$

has no zeros in the nondiagonal entries. That is, for any *i* ≠ *j*, there is a walk from vertex *i* to vertex *j*.

This completes the proof that treatment vertices *i* and *j* are connected directly or indirectly if and only if the entry (*C*_*n* − 1_)_*ij*_, and thus *I*(*C*_*n* − 1_)_*ij*_ is nonzero.

## APPENDIX B

### R CODE TO PERFORM NETWORK CONNECTEDNESS TEST AND RETURN DISTANCE MATRIX

# *Function to test whether an evidence network is connected and generate the distance matrix*.

# *The input tr.matrix is same format as treatment matrix “t” used in the NICE DSU TSD 2 WinBUGS code for network meta‐analysis*.

# *An alternative to supplying tr.matrix is to supply vectors t1 and t2. These include one entry for each arm of the trials and t1 represents a list of trial IDs while t2 represents a list of treatments*.

*require (expm)*.

*test.connectedness.fun<−function (tr.matrix = NULL,t1 = NULL,t2 = NULL,nameoftreatments = NULL,sort.matrices = TRUE)*.

*{*

# *tr.matrix is a matrix of rows corresponding to trials and columns*

# *corresponding to treatments*

*if (is.null (tr.matrix) & (is.null(t1)|is.null(t2))){*

*stop(“Must provide tr.matrix or both t1 and t2 vectors”)*

*}*

# *Vectors t1 and t2 include one entry for each arm of the trials*

# *t1 represents a list of trial IDs*

# *t2 represents a list of treatments*

# *These will be constructed if not provided*

# *nameoftreatments converts the numbers of t2 into treatment names*

# *Construct the t1 and t2 vectors if only treatment matrix is provided*

*if (is.null(t1)|is.null(t2))*

*{*

*x < −tr.matrix*

*for(i in 1:dim(x) [1])*

*{*

*x [i,] < −i*

*}*

*t1 < −c(x[!is.na (tr.matrix)])*

*t2 < −c (tr[!is.na (tr.matrix)])*

*}*

*# Build a matrix of 1 s and 0 s representing direct evidence connections*

*n.trials<−length (unique(t1))*

*n.treatments<−length (nameoftreatments)*

*if (is.null (nameoftreatments))n.treatments<−length (unique(t2))*

*adjacency.matrix<−matrix(0,nrow = n.treatments,ncol = n.treatments)*

*t2 = as.numeric (as.factor(t2))*

*for (trial.i in unique(t1))*

*{*

*adjacency.matrix [t2[t1==trial.i],t2[t1==trial.i]] < −1*

*}*

*# Set the diagonal to 0*

*diag (adjacency.matrix) < −0*

*# Sum the powers of the adjacency matrix to calculate the indirect connection matrix*

*treatment.indirect.connection<−adjacency.matrix*

*for(i in 2:(n.treatments‐1))*

*{*

*treatment.indirect.connection<−treatment.indirect.connection+adjacency.matrix%^%i*

*}*

# *Set all non‐zero elements to 1*

*treatment.indirect.connection<−treatment.indirect.connection! = 0*

# *Convert to numeric*

*treatment.indirect.connection<−matrix (as.numeric (treatment.indirect.connection),ncol = n.treatments)*

# *Calculate the distance matrix*

*distance.matrix<−adjacency.matrix*

*for(i in 2:(n.treatments‐1))*

*{*

*A.im1 < −adjacency.matrix*

*for(j in 1:(i‐1))*

*{*

*A.im1 < −A.im1 + adjacency.matrix%^%j*

*}*

*A.i < −A.im1 + adjacency.matrix%^%i*

*# Set non‐zero entries to 1*

*A.i [A.i! = 0] < −1*

*A.im1[A.im1! = 0] < −1*

*distance.matrix<−distance.matrix+i*(A.i‐A.im1)*

*}*

# *Set the diagonal to zero*

*diag (distance.matrix) < −0*

# *Name the rows and columns after the treatments*

*if(!is.null (nameoftreatments)){*

*rownames (distance.matrix) < −colnames (distance.matrix) < −rownames (adjacency.matrix) < −colnames (adjacency.matrix) < −rownames (treatment.indirect.connection) < −colnames (treatment.indirect.connection) < −nameoftreatments*

*}else{*

*warning(“No nameoftreatments provided”)*

*}*

# *Sort the matrices by treatment names?*

*if (sort.matrices==TRUE)*

*{*

# *Sort the entries in the matrices*

*adjacency.matrix<−adjacency.matrix [order (as.numeric (rownames (adjacency.matrix))),order (as.numeric (rownames (adjacency.matrix)))]*

*treatment.indirect.connection<−treatment.indirect.connection [order (as.numeric (rownames (treatment.indirect.connection))),order (as.numeric (rownames (treatment.indirect.connection)))]*

*distance.matrix<−distance.matrix [order (as.numeric (rownames (distance.matrix))),order (as.numeric (rownames (distance.matrix)))]*

*}*

*return (list(“adjacency.matrix” = adjacency.matrix,”indirect.connection.matrix” = treatment.indirect.connection,”distance.matrix” = distance.matrix))*

*}*

## APPENDIX C

### R CODE TO COLLECT INDIRECT CONNECTION MATRIX INTO CONNECTED COMPONENTS

# Recursive function to collect/sort the indirect connection and step matrices.

# into connected components.

# Simple function to collect matrices from 1st index.

# Used recursively in collect.matrix.

collect.submatrix<−function (collected.matrix).

{

# Indices of treatments connected and not connected to first treatment

collected.treatments<−c (which (collected.matrix[1,] > 0),which (collected.matrix[1,]==0))

# Split matrix into treatments connected and disconnected to first treatment

collected.matrix<−collected.matrix [collected.treatments,collected.treatments]

}

# Function to collect elements of a matrix into block‐diagonal form.

collect.matrix<−function (collected.matrix).

{

# Slightly different for first treatment.

collected.matrix<−collect.submatrix (collected.matrix).

for(j in 2:(dim (collected.matrix)[2]‐1)).

{

# If treatment is not connected to earlier treatment then more sorting required

if (collected.matrix [j,j‐1]==0)

{

# Sort the sub‐matrix starting at current treatment

temp.collected.matrix<−collect.submatrix (collected.matrix [j:n.treatments,j:n.treatments])

# Replace unsorted sub‐matrix with sorted sub‐matrix

collected.matrix [j:n.treatments,j:n.treatments] < −temp.collected.matrix

rownames (collected.matrix)[j:n.treatments] < −rownames (temp.collected.matrix)

colnames (collected.matrix)[j:n.treatments] < −rownames (temp.collected.matrix)

}

}

return (collected.matrix).

}

## APPENDIX D

### STATA CODE TO PERFORM NETWORK CONNECTEDNESS TEST, RETURN DISTANCE MATRIX AND IDENTIFY CONNECTED COMPONENTS

Suppose we have data from the network illustrated in Figure 2(a) in long format (i.e. one record per arm per trial) with binary outcome. Variable *trial* identifies the trial, *trt* identifies the treatment, *d* gives the number of events and *n* gives the number of patients. Then the standard command to set up the network is.

network setup d n, studyvar (trial) trtvar (trt).

which reports (among other statistics).

Components: 2 (disconnected).

The nature of the network can be understood by printing four matrices which are computed by *network setup*, provided a *network* version 1.2.2 (dated 21dec2015) or later is used. (To get this, enter STATA and type *net from http://www.mrc‐bsu.cam.ac.uk/IW_Stata/meta*.) Below is the STATA log:

. matrix list network_adjacency//the adjacency matrix.

symmetric network_adjacency.[8,8]

1 2 3 4 5 6 7 8.

1 0

2 1 0.

3 0 1 0.

4 0 0 0 0.

5 0 1 0 1 0.

6 0 0 0 0 0 0.

7 0 0 0 0 0 1 0.

8 0 0 0 0 0 0 1 0

. matrix list network_indirect_connection//the indirect connection matrix.

symmetric network_indirect_connection.[8,8]

1 2 3 4 5 6 7 8.

1 1

2 1 1.

3 1 1 1.

4 1 1 1 1.

5 1 1 1 1 1.

6 0 0 0 0 0 1.

7 0 0 0 0 0 1 1.

8 0 0 0 0 0 1 1 1

. matrix list network_distance//the distance matrix.

symmetric network_distance.[8,8]

1 2 3 4 5 6 7 8.

1 0

2 1 0.

3 2 1 0.

4 3 2 3 0.

5 2 1 2 1 0.

6 0 0 0 0 0 0.

7 0 0 0 0 0 1 0.

8 0 0 0 0 0 2 1 0

. matrix list network_components//assigns treatments to components.

network_components.[8,2]

c1 c2.

1 1 0.

2 1 0.

3 1 0.

4 1 0.

5 1 0.

6 0 1.

7 0 1.

8 0 1.
